# Das modifizierte Treat-and-Extend-Schema mit Injektionsblöcken in der IVOM-Therapie: Retrospektive Analyse aus der täglichen klinischen Anwendung

**DOI:** 10.1007/s00347-020-01218-y

**Published:** 2020-09-07

**Authors:** Patricia Take, Carolin Alisa Dittmann, Laura Mackerodt, Josep Callizo, Nina-Antonia Striebe, Hans Hoerauf, Nicolas Feltgen, Sebastian Bemme

**Affiliations:** Augenklinik der Universitätsmedizin, Georg-August-Universität Göttingen, Robert-Koch-Str. 40, 37075 Göttingen, Deutschland

**Keywords:** Treat-and-Extend, Anti-VEGF, Altersbedingte Makuladegeneration, Diabetisches Makulaödem, Retinaler Venenverschluss, Treat and extend, Anti-VEGF, Age-related macular degeneration, Diabetic macular edema, Retinal vein occlusion

## Abstract

**Hintergrund:**

Die regelmäßige Therapie mittels intravitrealer operativer Medikamentenapplikation (IVOM) und die damit verbundenen Kontrolluntersuchungen stellen für viele Patienten eine große Herausforderung dar, die bis zum Behandlungsabbruch führen kann. Das modifizierte blockweise *Treat-and-Extend*(TAE)-Schema verfolgt das Ziel, mit möglichst wenigen Kontrollvisiten stabile Netzhaut- und Visusbefunde zu erreichen und dadurch die Belastung der Patienten zu minimieren.

**Methoden:**

Diese monozentrische retrospektive Studie untersuchte Behandlungsverläufe von insgesamt 387 Patienten mit neovaskulärer altersbedingter Makuladegeneration (AMD), diabetischem Makulaödem (DMÖ), Venenastverschluss (VAV) und Zentralvenenverschluss (ZVV), bei denen das TAE-Regime jeweils in 3er-Blöcken angewendet wurde. Primärer Endpunkt war das Erreichen eines Injektionsintervalls von 12 Wochen.

**Ergebnisse:**

Durch die blockweise applizierte IVOM konnte die Netzhautdicke signifikant reduziert und der Visus verbessert werden. Über die verschiedenen Indikationen konnte im Mittel ein Behandlungsintervall von 2 Monaten erreicht werden.

**Schlussfolgerung:**

Ein in 3er-Blöcken angepasstes TAE-Schema im Rahmen der IVOM kann bei reduzierter Patientenbelastung zu stabilen Netzhaut- und Visusbefunden führen.

**Zusatzmaterial online:**

Zusätzliche Informationen sind in der Online-Version dieses Artikels (10.1007/s00347-020-01218-y) enthalten.

## Abstract (en)

**Background:**

Regular treatment with intravitreal operative medication injections (IVOM) and the associated frequent check-ups are a major challenge for many patients, which can lead to treatment discontinuation. The aim of the modified treat and extend (TAE) in blocks scheme is to achieve stable retinal and visual outcomes with as few control visits as possible and thus to minimize the burden for the individual patient.

**Methods:**

This monocentric retrospective study examined the treatment courses of 387 patients with neovascular age-related macular degeneration, diabetic macular edema and retinal vein occlusion, who were treated by three blocks of intravitreal injections in the TAE regimen. The primary endpoint was achieving an injection interval of 12 weeks.

**Results:**

Intravitreal injections in TAE blocks significantly reduced retinal thickness and improved visual acuity. On average, a treatment interval of 2 months was achieved across the various indications.

**Conclusion:**

Intravitreal injections in TAE blocks of three injections can reduce patients’ burden and lead to stable retinal and visual results.

Die intravitreale operative Medikamentenapplikation (IVOM) stellt für den Patienten eine persönliche und für den Klinikalltag eine organisatorische Herausforderung dar. Während in den Zulassungsstudien von Anti-VEGF-(Vascular Endothelial Growth Factor-)Präparaten (Phase III) für alle Indikationen eine deutliche Steigerung der zentralen Sehschärfe erreicht und über 1 bis 2 Jahre stabilisiert wurde, konnte in Phase IV der anfänglich erzielte Visuserfolg nicht gehalten werden [5, 14, 29]. In Anwendungsbeobachtungen kam es zudem zu vorzeitigen Therapieabbrüchen aufgrund der Entfernung zur Klinik, des Krankheitsverständnisses, der Morbidität der meist älteren Patienten und fehlender Unterstützung aus dem familiären Umfeld [8, 30].

Auch Daten der Göttinger Augenklinik aus den Jahren 2013 und 2014 zeigten eine Rate von 64 % Behandlungsabbrüchen bei Anwendung des PRN-Schemas innerhalb eines Jahres. In der Folge wurde das Behandlungsschema in ein *Treat-and-Extend*(TAE)-Schema verändert. Dabei wurde nicht das TAE-Regime mit Einzelinjektionen und 2‑wöchiger Verlängerung oder Verkürzung des Intervalls gewählt, sondern eine Modifikation, die von Mantel et al. für die Behandlung der exsudativen AMD beschrieben wurde [18]. Diese besteht aus einem initialen Injektionszyklus von 4 IVOM alle 4 Wochen, gefolgt von 3er-Blöcken mit Änderung des Injektionsabstands zwischen den Blöcken um jeweils 2 Wochen (Abb. 1). Bei der Untersuchung am Tag der jeweils letzten Injektion eines Blocks wird das weitere Vorgehen entschieden: Konnte die Krankheitsaktivität vollständig zurückgedrängt und ein trockener Befund stabilisiert werden, wird das Blockintervall um 2 Wochen verlängert (in Abb. 1 als „Stabilisierung“ definiert). Unveränderte subretinale Flüssigkeit sowie degenerative Zysten wurden dabei als inaktive Zeichen toleriert. Bei persistierender Aktivität oder Befundverschlechterung wie Blutungen, Zunahme der intra- oder subretinalen Flüssigkeit wird das Intervall um 2 Wochen verkürzt (in Abb. 1 als „Aktivität“ definiert). Durch diese individualisierte Therapie soll eine optimale Behandlung, d. h. ein stabiler Netzhautbefund mit einer möglichst geringen Belastung für den einzelnen Patienten, erreicht werden. Die Behandlung wurde beendet, wenn das Behandlungsintervall auf 12 Wochen ausgedehnt werden konnte. Bezogen auf ihre Wirksamkeit, sind die in dieser Studie eingesetzten Inhibitoren (Ranibizumab, Aflibercept und Bevacizumab) aufgrund der aktuellen Studienlage in gleicher Dosierung als gleichwertig effektiv anzusehen [17, 20, 24]. Ihre Wirkung ist sowohl für die AMD [2, 5, 14, 17], das diabetische Makulaödem [4] als auch für das Makulaödem nach retinalem Venenverschluss [3, 24] durch Studien bestätigt.

**Figure 1:**
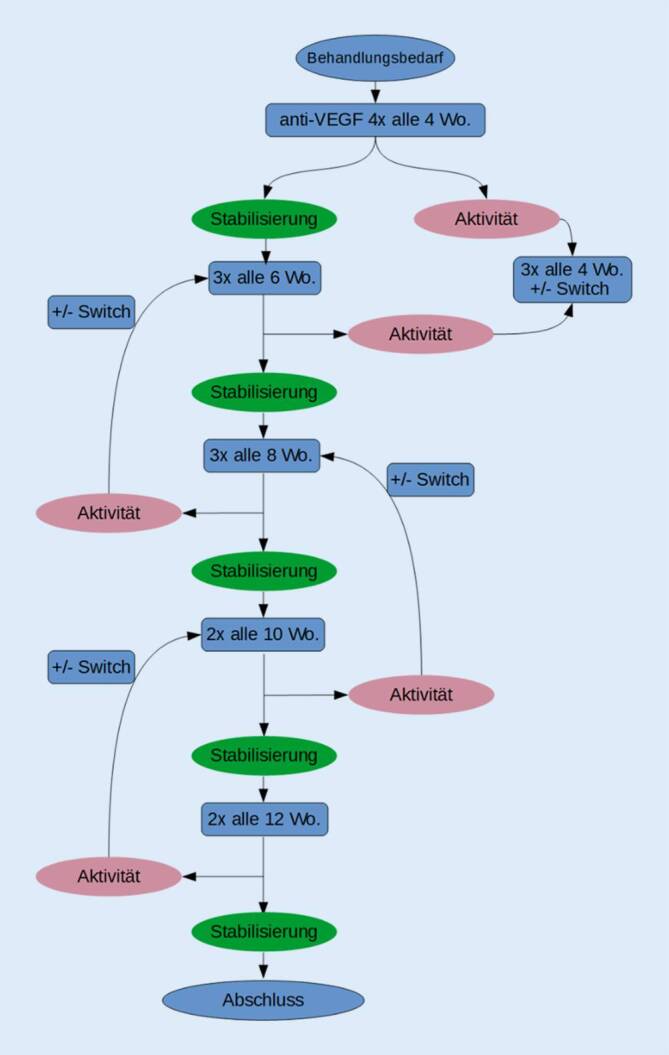

Ziel der vorliegenden Studie war die retrospektive Analyse des modifizierten TAE-Schemas. Die zentrale Fragestellung untersuchte, bei wie vielen Patienten ein behandlungsfreies Injektionsintervall von 3 Monaten und damit ein Therapieabschluss erreicht wurde.

## Material und Methoden

### Studienkohorte

In diese retrospektive monozentrische Studie wurden Patienten eingeschlossen, die im Zeitraum zwischen 01.10.2015 bis zum 20.06.2018 ihre erste IVOM an der Augenklinik der Universitätsmedizin Göttingen erhielten. Folgende Diagnosen wurden eingeschlossen: chorioidale Neovaskularisation bei altersabhängiger Makuladegeneration (CNV bei AMD), Makulaödem bei retinalem Zentralvenen- und Venenastverschluss (ZVV und VAV) und Makulaödem bei diabetischer Makulopathie (DMÖ). Es musste Behandlungsnaivität oder eine mindestens 6‑monatige Behandlungspause mit VEGF-Inhibitoren sowie der Nachweis eines Makulaödems in der Spectral-Domain-optischen Kohärenztomographie (SD-OCT) vorliegen.

Ausschlusskriterien waren eine aktive intraokulare Entzündung, Zustand nach Vitrektomie oder intravitrealer Behandlung mit Steroiden, eine Kataraktextraktion innerhalb von 3 Monaten vor der ersten Injektion sowie ausgeprägte Medientrübung und andere Formen einer Makulopathie.

### Ausgewertete Parameter und Endpunkte

Die Datenerfassung erfolgte mithilfe der Dokumentationssoftware *IVI-Manager* (Version 10.0, Universitätsmedizin Göttingen, Göttingen, Deutschland). Erfasst wurden die Anzahl der Injektionen und Kontrollvisiten, der Abstand zwischen Diagnosestellung und erster Injektion sowie der Abstand zwischen den einzelnen Injektionen im Zeitraum 01.10.2015 bis zum 20.06.2018. Die in dieser Arbeit angegebene Behandlungsdauer entspricht dem Zeitraum von erster bis letzter IVOM im Studienzeitraum. Sie spiegelt damit nicht die tatsächliche Behandlungsdauer des einzelnen Patienten wider, sondern entspricht der Beobachtungszeit in dieser Studie. Zur Ermittlung des maximal erreichten Injektionsintervalls wurden nur Intervalle einbezogen, die höchstens 2 Wochen länger als das jeweils vorherige und kleiner als 14 Wochen waren, um diesen Parameter nicht durch Behandlungspausen oder -verzögerungen zu verfälschen. Die Injektionsintervalle wurden dafür auf die ganze Wochenanzahl gerundet. Primärer Endpunkt war der Anteil der Patienten, die einen Injektionsabstand von 12 Wochen erreichten. Hierfür wurden nur die Patienten einbezogen, die ihre erste Injektion bis einschließlich 26.10.2016 erhielten und denen es somit rein rechnerisch überhaupt möglich war, ein 12-Wochen-Intervall zu erreichen. Zur Berechnung des Anteils der Patienten, die ein 10-Wochen-Intervall erreicht haben, wurde vergleichbar vorgegangen, sodass nur Patienten, die bis einschließlich 29.03.2017 ihre erste IVOM erhalten hatten, in die Berechnung einbezogen wurden. Weitere sekundäre Zielparameter sind in der Tab. 1 dargestellt. OCT-morphologische Parameter wurden in Analogie zum ORCA-Modul der OCEAN-Studie erhoben, wobei die Netzhautdicke zwischen der Bruch-Membran als äußere und der inneren Grenzmembran (ILM) als innere Grenze gemessen wurde [11].

**Table 1:** 

*Primärer Endpunkt: Anteil der Patienten mit einem injektionsfreien Intervall von 12 Wochen*
*Sekundäre Zielparameter*
Anzahl der IVOM
Dauer von Diagnosestellung in der Klinik bis zum Behandlungsbeginn
Anzahl der verwendeten Medikamente pro Diagnose
Anzahl der Kontroll-Wirkungs-Untersuchungen (vor der IVOM) zur Therapieplanung
Anteil der Patienten, die einen Injektionsabstand von 10 Wochen erreichen
OCT-morphologische Parameter nach dem ORCA-Modul der OCEAN-Studie
Bestkorrigierter Visus

### Statistische Auswertung

Die statistische Auswertung erfolgte mittels Wilcoxon-Vorzeichen-Rang-Test und der Software Graphpad Prism (GraphPad Software, La Jolla, USA).

## Ergebnisse

### Epidemiologie und verwendete Medikamente

Im Untersuchungszeitraum wurden 1136 Datensätze ermittelt. Nach Ausschluss konnten die Daten von insgesamt 387 Patienten im angegebenen Zeitraum ausgewertet werden: 254 Patienten hatten eine AMD, 62 Patienten ein DMÖ, 40 Patienten einen VAV und 31 Patienten einen ZVV. AMD-Patienten waren am ältesten, DMÖ-Patienten am jüngsten (Tab. 2). Am häufigsten wurde Aflibercept injiziert (*N* = 2187), gefolgt von Ranibizumab (*N* = 951) und Bevacizumab (*N* = 796). Dabei hat sich der prozentuale Anteil der verschiedenen VEGF-Inhibitoren in den Untergruppen (12 Wochen erreicht, 10 Wochen erreicht) nicht verändert.

**Table 2:** 

Erkrankung	AMD	DMÖ	VAV	ZVV
Anzahl Patienten	*N* = 254	*N* = 62	*N* = 40	*N* = 31
Alter bei erster Injektion (Jahre)	78,1 ± 8,0 79,0	62,4 ± 13,9 64,5	68,0 ± 12,2 71,5	73,3 ± 9,5 75,0
Geschlecht (w/m)	147/107	17/45	23/17	13/18
Injektionsseite (RA/LA)	112/142	32/30	19/21	17/14
Medikamente (Anzahl *N*)				
Ranibizumab	718	55	81	97
Bevacizumab	540	74	109	73
Aflibercept	1348	433	243	163
*Injektionen gesamt*	*2606*	*562*	*433*	*333*

Anzahl und Alter (Mittelwert ± Standardabweichung, Median) eingeschlossener Patienten mit Verteilung bezüglich Geschlecht und Injektionsseite. Gesamtzahl ausgewerteter Injektionen und Anzahl der dafür verwendeten Medikamente je Erkrankung

### Häufigkeit der Injektionen und Kontrollen

**Figure 2:**
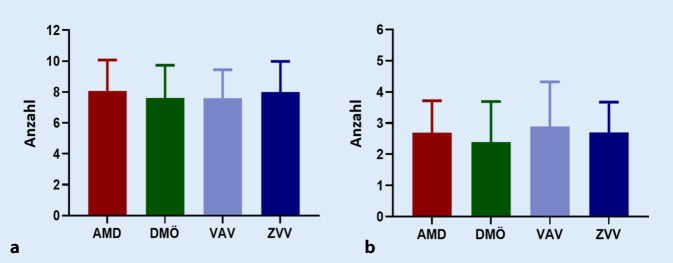

Insgesamt wurden 3934 IVOM durchgeführt. Die Behandlungsdauer war aufgrund des retrospektiven Designs unterschiedlich und betrug im Median 58 (AMD), 48 (DMÖ), 59 (VAV) und 61 (ZVV) Wochen. Die DMÖ- und VAV-Patienten erhielten mit durchschnittlich 7,6 IVOM die wenigsten Injektionen im ersten Jahr, die AMD-Patienten wurden mit 8,1 IVOM am häufigsten injiziert (Abb. 2a). Alle Patienten erhielten im Schnitt 2 bis 3 Kontrollen im ersten Jahr (Abb. 2b) bzw. eine Kontrolle nach ca. jeder 3. Injektion. Eine dokumentierte Behandlung über 12 Monate, bezogen auf die Patienten, die mindestens 1 Jahr vor Studienende ihre erste Injektion erhielten, lag bei insgesamt 70,7 % (AMD 73,2 %; DMÖ 61,4 %; VAV 62,9 %; ZVV 78,3 %). Behandlungsabbruch oder Übernahme der Behandlung durch niedergelassene Kollegen wurden nicht dokumentiert.

### Injektionsintervalle und primäres Zielkriterium

**Figure 3:**
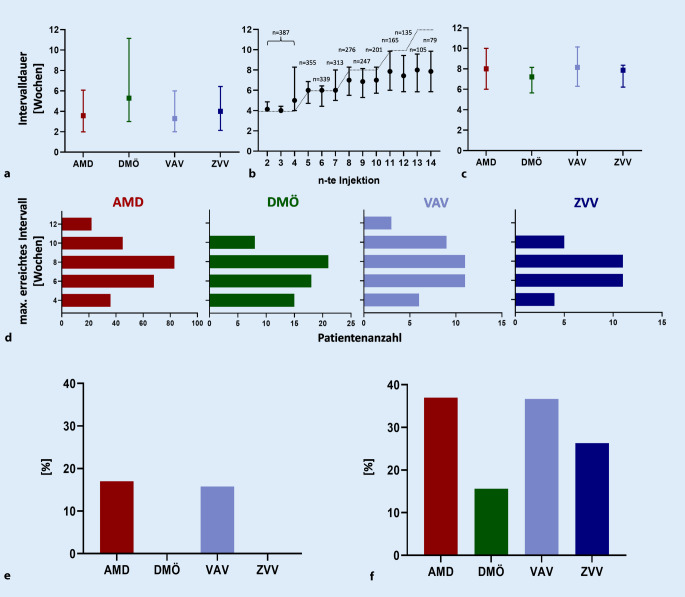

Die Dauer von der ersten Vorstellung in der Klinik bis zur ersten IVOM betrug im Median 3,3 (VAV) bis 5,3 (DMÖ) Wochen (Abb. 3a).

Für alle Indikationen konnten die Abstände ausgedehnt werden, stagnierten aber nach der 10. Injektion (Abb. 3b). Das maximal erreichte Injektionsintervall betrug zwischen 7,2 (DMÖ) bis 8,1 (VAV) Wochen (Abb. 3c). Die Abb. 3d stellt die Verteilung der absoluten Patientenzahlen auf die maximal erreichten Injektionsintervalle für die jeweilige Erkrankung dar. Hierbei wurden nur Intervalle, die höchstens 2 Wochen länger als das jeweils vorherige, einbezogen. Bei 2363 von 3160 Folgeinjektionen (74,8 %; gezählt ab der 3. Injektion) war der Injektionsabstand maximal 2 Wochen länger oder kürzer als das vorherige Intervall. In 15,9 und 9,4 % der Injektionen wurde das Injektionsintervall um mehr als 2 Wochen verlängert bzw. verkürzt.

Insgesamt hatten rechnerisch 100 AMD-, 18 DMÖ-, 19 VAV- und 11 ZVV-Patienten die Möglichkeit, eine Injektion nach 12 Wochen zu erhalten und damit das primäre Zielkriterium zu erfüllen; 17 % der AMD-Patienten und 16 % der VAV-Patienten erreichten dieses 12-Wochen-Intervall. Kein DMÖ- oder ZVV-Patient konnte auf 12 Wochen extendiert werden (Abb. 3e). Ein 10-Wochen-Intervall erreichten jeweils 37 % (AMD/VAV), 26 % (ZVV) und 16 % (DMÖ) der Patienten (Abb. 3f).

### Visus und Netzhautdicke

**Figure 4:**
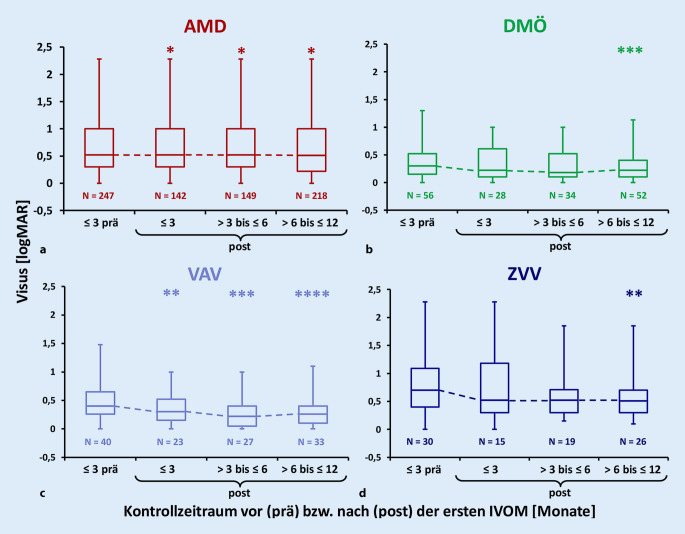

Im Beobachtungszeitraum kam es zu einer Visusverbesserung über alle Indikationen (1,2 Zeilen, entsprechend 6 Buchstaben auf der ETDRS-Tafel, *p* < 0,0001) (Abb. 4). Die Sehschärfe blieb bei AMD-Patienten über 12 Monate im Mittel stabil, bei DMÖ-Patienten verbesserte sich der Visus um 4 Buchstaben, und VAV- und ZVV-Patienten gewannen 7 bzw. 10 Buchstaben.

**Figure 5:**
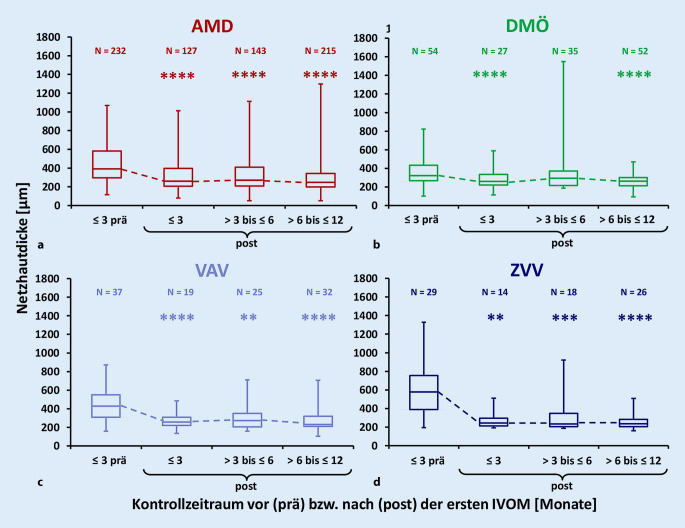

Insgesamt wurden 1085 OCT-Untersuchungen ausgewertet. Die Netzhautdicke zeigte über alle Indikationen im Median eine signifikante Reduktion von 397,0 µm (≤3 Monate vor erster IVOM) auf 243,3 µm (>6 ≤ 12 Monate nach erster IVOM) (*p* < 0,0001). Die stärkste Reduktion zeigte sich bei ZVV mit 342,5 µm, gefolgt von VAV mit 196,3 µm, AMD mit 146 µm und DMÖ mit 60 µm (Abb. 5).

## Diskussion

Die vorliegende Studie untersuchte erstmals die Ergebnisse eines modifizierten TAE-Schemas über die Indikationen AMD, DMÖ, VAV und ZVV und überprüft die Umsetzbarkeit der Strategie in den Alltag.

### Behandlungsintensität

Die Anzahl an Injektionen entspricht mit im Median 8 bis 9 IVOM im ersten Jahr in etwa der Therapie nach konventionellem Schema [9, 10, 18, 23]. Wie wichtig eine konsequente und v. a. initial intensive Therapie für das funktionelle Ergebnis ist, wurde immer wieder gezeigt [11, 28]. Dies ist mit dem modifizierten TAE-Schema realisierbar. Die Anzahl der Kontrollen zur weiteren Therapieplanung mit 3 Visiten im ersten Jahr bei allen Indikationen erfüllt die Erwartungen an einen extendierenden 3er-Block und liegt unter der Visitenzahl bei PRN- oder TAE-Schemata mit Einzelinjektionen [1, 12, 28]. In der Studie von Mantel et al. waren im ersten Behandlungsjahr 3,97 Visiten bei AMD-Patienten erforderlich [18]. Die reduzierte Belastung für die Patienten ist vereinbar mit einer vergleichsweise zufriedenstellenden Patientenadhärenz von 70,7 % über mehr als 12 Monate in der aktuellen Studie.

In unserer Analyse erreichten AMD-Patienten mit einem 8‑wöchigen Therapieintervall ein ähnliches Resultat im Vergleich zu Mantel et al. mit ca. 1,97 Monaten [18]. Das erreichte Intervall von 7,2 Wochen bei DMÖ entspricht ebenfalls der Literatur [10]. Für ZVV-Patienten liegt der Wert mit 7,9 Wochen etwas unter dem durchschnittlich erreichten Intervall anderer Arbeiten [21].

Ein Abstand von 12 Wochen – und damit ein in unserem Haus definiertes Therapieende – konnte bei AMD-Patienten in 17 % und bei VAV-Patienten in 16 % der Fälle erzielt werden. Studien anderer Arbeitsgruppen erreichten eine Quote zwischen 18 und 27 % für AMD und 65,6 % für VAV [1, 2, 13, 18]. Kein DMÖ- oder ZVV-Patient erreichte nach unserem Schema ein 12-Wochen-Intervall. Andere Studien berichten das Erreichen des 12-Wochen-Intervalls nach konventionellem TAE bei ZVV und DMÖ in 29,4 % bzw. 64–73,9 % [7, 21, 22].

In unserer Studie konnte kein Einfluss der Medikamentenwahl auf die Intervalllänge festgestellt werden. Die verschiedenen Substanzen waren in allen Intervallgruppen gleich verteilt. Daher sind neue Anti-VEGF-Substanzen mit verlängerter Wirkung wünschenswert. Besondere Aufmerksamkeit erfordert zudem der große Abstand zwischen Indikationsstellung und erster Injektion, insbesondere bei Diabetikern. Hierbei wird klar, dass bei einer Verzögerung um ca. 3 bis 5 Wochen die Empfehlungen der OCEAN-Studie (Behandlungsbeginn innerhalb von 2 Wochen) trotz aller Anstrengungen außerhalb klinischer Studien nicht eingehalten werden konnten [28]. Unsere Klinik reagierte auf dieses Ergebnis mit erneuter Aufklärung aller Mitarbeiter im Hause sowie der Einrichtung von Notfallterminen bei Erstindikation. Bezüglich der Folgeinjektionen zeigte sich, dass die vorgegebenen Zeitabstände bei einem Großteil der Injektionen eingehalten werden konnten. Die Injektionsintervalle waren in 74,8 % höchstens 2 Wochen länger oder kürzer als der vorherige Injektionsabstand gemäß der nach dem Schema maximal erlaubten Extension oder Verkürzung.

### Funktioneller und morphologischer Therapieerfolg

Aktuelle Daten einer prospektiven kanadischen Studie bestätigten, dass sich die Visusentwicklung bei TAE bei AMD nicht von monatlichen Injektionsgaben unterscheidet [15]. Inwieweit sich dies auch für ein TAE-Schema mit 3er-Blöcken übertragen lässt, wurde erstmalig in unserer Studie für die 4 häufigsten Indikationen untersucht. Die Sehschärfe verbesserte sich um 6 Buchstaben über alle Indikationen, wobei Unterschiede in den einzelnen Diagnosen auffallen. Der Zugewinn von 7 und 10 Buchstaben bei VAV und ZVV ist mit Untersuchungen vergleichbar, die einen Visusgewinn von 6 bis 22,2 Buchstaben bei konventionellem TAE zeigten [9, 16, 21, 23]. Patienten mit DMÖ verbesserten sich in unserer Studie um 4 Buchstaben, andere Studien beschrieben eine Verbesserung von 4,3 bis 9,1 Buchstaben [7, 22]. Bei AMD-Patienten konnte der Visus in unserer Studie immerhin stabilisiert werden. In der AURA-Studie betrug der Zugewinn bei AMD 2,4 Buchstaben ETDRS innerhalb eines Jahres [12], in der OCEAN-Studie lag er bei 5 Buchstaben ETDRS innerhalb der ersten 3 Monate [28], Mantel et al. beschreiben einen Zugewinn von 9,8 Buchstaben ETDRS [18]. Unsere Patienten wurden unabhängig vom Ausgangsvisus eingeschlossen, sodass möglicherweise durch den Einschluss von AMD-Patienten mit Fibrose- und Atrophieveränderungen auch schwere Krankheitsverläufe einbezogen wurden. Das modifizierte TAE-Schema konnte die Netzhautdicke signifikant reduzieren, wobei die Ergebnisse im Vergleich mit konventionellen TAE-Schemata diagnoseübergreifend in ähnlichen Bereichen lagen [2, 7, 9, 16, 18, 21–23, 26].

### Mögliche Nachteile des modifizierten TAE-Schemas im 3er-Block

Die IVOM-Applikation in Blöcken führt zu einem recht langen Intervall zwischen den Visus- und OCT-Kontrolluntersuchungen, was die Patientenbelastung reduziert. Andererseits könnte bei der Wahl eines zu langen Intervalls bei 3 konsekutiven IVOM ohne Kontrolle ein Rezidiv zu lange unerkannt bleiben. Bei AMD könnte dies einen irreversiblen Verlust darstellen, diese Befürchtung konnte in unserer Studie nicht bestätigt werden. Letztlich bleibt bei dem vorliegenden Schema noch die Frage der Überdosierung zu diskutieren, da die Gabe einer 3er-Serie ggf. nicht bei jedem Patienten erforderlich wäre, sondern eine Extension auch mit weniger Injektionen zügiger erreicht werden könnte. Dem steht die geringe Anzahl an Kontrollen gegenüber, die vermutlich maßgeblich zu einer hohen Adhärenz beiträgt und somit wiederum einem Therapieabbruch und damit verbundener Unterbehandlung vorbeugt. Die Problematik einer Unterbehandlung ist in zahlreichen Studien belegt [6, 12, 19, 27]. Betrachtet man zudem die sehr geringe Komplikationsrate der intravitrealen Injektion, scheint das Risiko einer möglichen Überbehandlung im Vergleich zum potenziellen Schaden bei Unterbehandlung kaum ins Gewicht zu fallen [27]. Dies wird auch in der neuen DOG-Stellungnahme zur Behandlung des diabetischen Makulaödems mit der Empfehlung von initial 6 Injektionen im Abstand von 4 Wochen deutlich [25].

### Limitationen der Studie

Bei der Interpretation der Ergebnisse muss beachtet werden, dass von allen im Beobachtungszeitraum behandelten Patienten nur etwas mehr als ein Drittel in die Studie eingeschlossen werden konnte und die Nachbeobachtungszeit relativ kurz gefasst war. Die ursprünglich erwartete Gruppengröße lag deutlich höher, aufgrund der Regeln für eine ausschließliche intravitreale Behandlung mit Anti-VEGF-Präparaten und vollständige Dokumentation mussten aber einige Patienten ausgeschlossen werden. Aufgrund der methodischen Anordnung hatte nur ein Teil der Patienten die Möglichkeit, ein 12-Wochen-Intervall überhaupt zu erreichen.

### Schlussfolgerung

Insgesamt kann das modifizierte TAE-Schema im 3er-Block diagnoseübergreifend dazu beitragen, bei reduzierter Anzahl von Kontrollvisiten den Visus zu stabilisieren und das Makulaödem zu verbessern, jedoch führt es in der Breite der Erkrankungen nicht zu einem im Mittel verlängerten Behandlungsintervall als andere Schemata.

## Fazit

- Das TAE-Schema im 3er-Block konnte bei reduzierter Patientenbelastung diagnoseübergreifend eine Visusstabilisierung und eine langfristig signifikante Reduktion der Netzhautdicke bewirken.
- Bei AMD- und RVV-Patienten konnte ein Behandlungsintervall von ca. 8 Wochen und bei DMÖ-Patienten ein Intervall von 7 Wochen erreicht werden.
- Es erreichten 17 % der AMD- und 16 % der VAV-Patienten ein 12-Wochen-Intervall.

## Caption Electronic Supplementary Material


