# Supplementary material for: The modified treat and extend scheme with injection blocks in intravitreal injection treatment: Retrospective analysis from the routine clinical application
Source: Ophthalmologe. 2020 Sep 7;118(6):578–86. [Article in German] doi: 10.1007/s00347-020-01218-y (PMC8187201; doi:10.1007/s00347-020-01218-y)
Supplement: Supplementary file 1 [file 347_2020_1218_MOESM1_ESM.docx]

| **Tab. S1: Anzahl der Injektionen und Kontrolluntersuchungen pro Patient im Behandlungszeitraum und im ersten Jahr** | | | | |
| --- | --- | --- | --- | --- |
| **Erkrankung** | **AMD** | **DMÖ** | **VAV** | **ZVV** |
| **Behandlungsdauer [Wochen]**  **(erste bis letzte IVOM)** | 60,7 ± 30,0  58,1 | 52,7 ± 29,2  47,8 | 68,7 ± 40,4  59,2 | 65,5 ± 33,6  60,9 |
| **Anzahl Injektionen** | 10,3 ± 4,0  10,0 | 9,1 ± 3,6  9,0 | 10,8 ± 4,8  10,5 | 10,7 ± 4,1  10,0 |
| **Anzahl Injektionen**  **(im ersten Jahr)** | **8,1 ± 2,0**  **9,0**  **(N = 198)** | **7,6 ± 2,1**  **8,0**  **(N = 44)** | **7,6 ± 1,9**  **8,0**  **(N = 35)** | **8,0 ± 2,0**  **8,0**  **(N=23)** |
| **Anzahl Kontrollen** | 3,6 ± 1,9  3,0 | 3,1 ± 1,8  3,0 | 4,1 ± 2,4  3,0 | 3,5 ± 2,0  4,0 |
| **Anzahl Kontrollen**  **(im ersten Jahr)** | **2,7 ± 1,0**  **3,0**  **(N = 198)** | **2,4 ± 1,3**  **2,0**  **(N = 44)** | **2,9 ± 1,4**  **3,0**  **(N = 35)** | **2,7 ± 1,0**  **3,0**  **(N=23)** |
| **Injektionen / Kontrollen** | 3,1 ± 1,2  3,0  (N=249) | 3,3 ± 1,3  3,4  (N=58) | 3,2 ± 1,5  3,0  (N=39) | 3,45 ± 1,9  3,2  (N=29) |

***Tab. S1:*** *Gesamtzahl der Injektionen und Kontrolluntersuchungen pro Patient im Behandlungszeitraum sowie Anzahl jeweils im ersten Jahr, insofern Datum der ersten IVOM nicht später als 1 Jahr vor Studienende. Angegeben sind jeweils Mittelwert ± Standardabweichung und der Median. Sofern nicht anders angegeben, beziehen sich die Daten auf die in Tab. 2 für jede Erkrankung angegebene Patientenanzahl N.*

| **Tab. S2: Injektionsintervalle** | | | | |
| --- | --- | --- | --- | --- |
| **Erkrankung** | **AMD** | **DMÖ** | **VAV** | **ZVV** |
| **Dauer von Diagnosestellung**  **bis zur ersten IVOM [Wochen]** | 6,4 ± 10,0  3,6 | 7,6 ± 14,2  5,3 | 5,0 ± 5,4  3,3 | 5,6 ± 7,2  4,0 |
| **maximal erreichtes IVOM-Intervall [Wochen]** | 8,0 ± 2,3  8,0 | 7,0 ± 2,0  7,2 | 8,2 ± 2,4  8,1 | 7,5 ± 1,8  7,9 |
| **primärer Endpunkt:**  **Anteil an Patienten mit**  **IVOM-Intervall ≥ 12 Wo** | **17,0 %**  **(17/100)** | **0,0 %**  **(0/18)** | **15,8 %**  **(3/19)** | **0,0 %**  **(0/11)** |
| **Anteil an Patienten mit**  **IVOM-Intervall ≥ 10 Wo** | 37,0 %  (61/165) | 15,6 %  (5/32) | 36,7 %  (11/30) | 26,3 %  (5/19) |

***Tab. S2:*** *Dauer von Diagnosestellung bis erste IVOM und maximal erreichtes IVOM-Intervall jeweils in Wochen. Angegeben sind jeweils Mittelwert ± Standardabweichung und der Median. Sofern nicht anders angegeben, beziehen sich die Daten auf die in Tab. 2 für jede Erkrankung angegebene Patientenanzahl N. Die Rate an Patienten, die ein maximales IVOM-Intervall von mind. 12 Wochen bzw. mind. 10 Wochen erreichten, bezieht sich nur auf Patienten, die die erste IVOM spätestens am 26.10.16 bzw. am 29.03.17 erhielten und damit ein max. Intervall von 12 bzw. 10 Wochen auch rechnerisch noch erreichen konnten.*
